# Microglia: The Hub of Intercellular Communication in Ischemic Stroke

**DOI:** 10.3389/fncel.2022.889442

**Published:** 2022-04-18

**Authors:** Yunsha Zhang, Lu Lian, Rong Fu, Jueling Liu, Xiaoqian Shan, Yang Jin, Shixin Xu

**Affiliations:** ^1^School of Integrative Medicine, Tianjin University of Traditional Chinese Medicine Tianjin, China; ^2^Medical Experiment Center, First Teaching Hospital of Tianjin University of Traditional Chinese Medicine Tianjin, China; ^3^Tianjin University of Traditional Chinese Medicine, Tianjin, China; ^4^National Clinical Research Center for Chinese Medicine Acupuncture and Moxibustion, First Teaching Hospital of Tianjin University of Traditional Chinese Medicine, Tianjin, China; ^5^Tianjin Key Laboratory of Translational Research of TCM Prescription and Syndrome, Tianjin, China

**Keywords:** microglia, intercellular communication, ischemic stroke, neuroinflammation, microglia polarization

## Abstract

Communication between microglia and other cells has recently been at the forefront of research in central nervous system (CNS) disease. In this review, we provide an overview of the neuroinflammation mediated by microglia, highlight recent studies of crosstalk between microglia and CNS resident and infiltrating cells in the context of ischemic stroke (IS), and discuss how these interactions affect the course of IS. The in-depth exploration of microglia-intercellular communication will be beneficial for therapeutic tools development and clinical translation for stroke control.

## Introduction

Microglia are initially termed “the third element of center nerve” and discovered as resident macrophages in the central nervous system (CNS). Microglia are primarily responsible for innate immunity in the brain and play a pivotal role in maintaining the homeostasis of the CNS through immune surveillance. As such, microglia can rapidly respond to the local milieu and drive acute inflammation as the defense and repair mechanism to maintain brain homeostasis. Although they are involved in immune responses within the CNS, they possess many additional capabilities beyond simple immune surveillance. Microglia, by themselves or interacting with other glial cells (astrocytes and oligodendrocytes), can instruct neurogenesis, regulate neural function, facilitate synapse formation and preserve the integrity of the blood-brain barrier (BBB; Nimmerjahn et al., 2005; Wake et al., 2009). Similarly, microglia react passively to brain pathology and play a more active role in the initiation and progression of many neurologic disorders, from neurodegeneration diseases to ischemic stroke (Wright-Jin and Gutmann, 2019).

Ischemic stroke (IS) is one of the leading causes of mortality and disability worldwide, and it is primarily caused by thromboembolic occlusion of a major artery that supplies the brain (Benjamin et al., 2018). Microglia are sensitive to ischemia and respond in minutes after IS onset. Microglia-mediated inflammation is a pathological hallmark of IS (Rodriguez-Gomez et al., 2020). Meanwhile, microglia can shape the fate of cell populations in CNS through cell-cell interactions. Hence, targeting microglia is of more interest in IS therapy. This review will briefly discuss the pathophysiology of microglia in IS, focusing on neuroinflammation, and then explore the communication mechanisms between reactive microglia and the CNS-resident cells and CNS-infiltrating cells.

## The Pathophysiology of Microglia in Ischemic Stroke

In response to hypoxia and ischemia-reperfusion injury in IS, the microglia undergo typical morphological and functional changes (Taylor and Sansing, 2013; Rodriguez-Gomez et al., 2020). In brief, the microglial reactivity was simplistic fitted into a bimodal scheme: the pro-inflammatory M1 and anti-inflammatory M2 phenotype. M2 is divided into M2a, M2b, and M2c. M2a is mainly involved in cell regeneration, and the other two types are primarily involved in phagocytosis and the removal of necrotic tissue (Chhor et al., 2013). M1 phenotype markers include CD16, CD32, CD86, major histocompatibility class II (MHC II), and inducible forms of nitric oxide synthase (iNOS). M1-microglia (M1-MG) secrete pro-inflammatory cytokines, such as TNF-α, IL-1β, and nitrogen monoxide (NO), and exacerbate inflammation and tissue injury. In contrast, M2-microglia (M2-MG) secrete anti-inflammatory cytokines, such as TGF-β, IL-4, IL-10, and growth factors such as vascular endothelial growth factor (VEGF), brain-derived neurotrophic factor (BDNF), platelet-derived growth factor (PDGF), suppress inflammation, and promote tissue recovery (Kanazawa et al., 2017).

Microglia could be activated within minutes following the onset of brain ischemia (Nakajima and Kohsaka, 2004) and play a significant role in phagocytosis within 3 days (Schroeter et al., 1997). Microglia exit morphologic diversity during brain ischemia, such as “stellate” microglia at 1 to 3 days and “amoeboid” microglia at 6 days after middle cerebral artery obstruction (MCAO; Schroeter et al., 1997). Consistent with these findings, another study showed that ramified microglia and “amoeboid” microglia surrounded intact and dying neurons after IS, respectively (Zhang et al., 1997; Perego et al., 2011).

The active microglia play biphasic roles in IS, depending on the opposite M1- and M2- phenotype. Existing studies show that M1-MG can be first detected in the ischemic penumbra at 6 h after cerebral ischemia and gradually extend to both ischemic penumbra and core after 24 h. An acute inflammatory process driven by M1-MG cleans the neural death debris and limits the expansion of infarct foci. Critical for regulating the inflammatory response, a secondary M2-like activation, followed by an M1-MG response, is essential for wound healing and suppression of inflammation. Studies detected Ym1 and CD206, characteristic markers of M2-MG, are exclusively expressed in the ischemic core in the acute phase of stroke (Lian et al., 2021). M2-MG, marked with CD206 or YM1, are observed as early as 6 h or 12 h and reach the peak approximately 24 h post-infarction. However, the dominant M2-MG in the core will gradually switch to M1-MG in the following 2 weeks (Perego et al., 2011; Taylor and Sansing, 2013). In principle, the M1-MG-induced pro-inflammatory response is not deleterious but necessary to maintain CNS homeostasis. However, for reasons not yet fully understood, M1-MG developed into a highly pro-oxidant phenotype, which contributes to the shift from acute to chronic inflammation aggravating neuronal death instead of repairing it.

In addition to regulating neuroinflammation, microglia are involved in other pathological events of stroke, such as activation of astrocytes, alteration of the blood-brain barrier, remyelination, and peripheral immune cell responses. And the communication between microglia and numerous cells is tightly focused on the microglial pro- and anti-inflammatory phenotypes.

## The Communication Between Microglia and Resident Cells in The Brain

**Figure 1:**
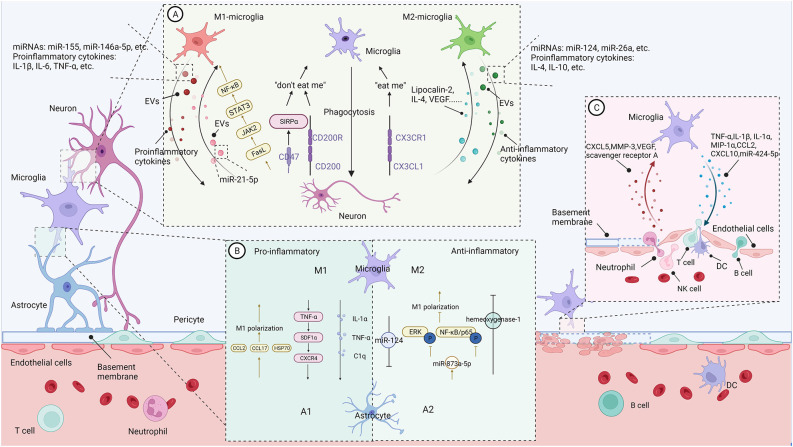
The communication between microglia and CNS-resident cells in the context of neuroinflammation (Figure created with BioRender.com). **(A)** Bidirectional microglia-neurons communication. Activated microglia generally perform phagocytosis to limit secondary neuronal damage caused by apoptotic or stressed neurons. Neurons release “eat me” or “don’t eat me” signals to modulate microglia’s phagocytosis. In addition, activated microglia increase the shedding of EVs containing pro- or anti-inflammatory cytokines and miRNAs, promoting or suppressing neural destruction. On the contrary, damaged neurons regulate microglial action and polarization through different signals. **(B)** Bidirectional microglia-astrocytes communication. Microglia with different phenotypes can modulate phenotypic transformation, proliferation, and glial scar formation of astrocytes through pro-inflammatory or anti-inflammatory signals. Similarly, astrocytes regulate microglial phenotypes and functions through their secreted molecules. **(C)** Bidirectional microglia-microvascular endothelial cells communication. Interactions between activated microglia and the endothelial cells of the BBB increase the permeability of the blood-brain barrier and promote leukocyte infiltration. In contrast, bidirectional communication between microglia and peripheral immune cells enhances central nervous system inflammation and promotes disease progression.

The modern concept of acute ischemic stroke highlights the role of the neurovascular unit (NVU) and emphasizes the importance of the dynamic interactions of endothelial cells, astrocytes, microglia, and neurons. Microglia, the pivotal immune sentinels, interact with other components in the NUV to form a complex network involved in the progression of IS (Figure 1).

### Neuron

In addition to their pivotal role as immune sentinels of the brain, emerging data suggest that microglia are critical to neuronal homeostasis. Meanwhile, neuronal activity is confirmed to be a driver of microglial action through “on” and “off ” signals. As such, most studies focus on the mechanisms of bidirectional microglia-neuron communication and demonstrate that there are a lot of cell-cell signals involved in indirect interaction (Marinelli et al., 2019; De Schepper et al., 2021) and direct interaction at somatic microglia-neuron junctions (Cserép et al., 2020, 2021).

### Microglia Shape the Fate of Neurons in Ischemic Stroke

As mentioned above, under the hypoxic environment following hypoperfusion, microglia are the first responders and then polarize toward M1- and M2-MG. Regardless of the functional phenotype, activated microglia generally perform phagocytosis to limit secondary neuronal damage caused by apoptotic or stressed neurons. These degenerated cells release “eat-me” signals to trigger microglia phagocytosis. Consistent with this motivation, during the acute phase of IS, lots of activated microglia accumulate mainly in the peri-infarct zone, where ischemia is less severe than in the infarct zone and neuronal cells are more inclined toward apoptosis. Moreover, these activated microglia can engulf infiltrating neutrophils for neuroprotective purposes (Neumann et al., 2008). The phagocytosis of microglia may serve either for killing cells (toxicity, M1) or scavenging debris (protection, M2c; David and Kroner, 2011). For instance, M1-MG may exhibit excessive phagocytosis, also called phagoptosis (Wang K. et al., 2021), eliminating viable neurons (Nehera et al., 2013; Brown and Neher, 2014) and live synapses (Shi X. et al., 2021) through multiple epidermal growth factor-like domains protein 10 (MEGF10) and tyrosine-protein kinase Mer (MERTK) in the post-stroke repair and remodeling stage. And thus exacerbating secondary damage after stroke.

The growing evidence indicated that extracellular vesicles (EVs) mediate microglia-neuron communication (Frühbeis et al., 2013). In response to pro-inflammatory stimuli, microglia increase the shedding of EVs containing pro-inflammatory cytokines and miRNAs, promoting neural destruction. Lipopolysaccharide (LPS) can stimulate microglia to release EVs enriched in IL-1β and miR-155 (Kumar et al., 2017), or TNF-α and IL-6 (Yang et al., 2018), contributing to pro-inflammatory signaling. Exosomes from BV2 microglia treated with α-syn contain TNF-α and MHC II proteins and increase neuronal apoptosis (Chang et al., 2013). Moreover, EVs from M1-MG enriched with miR-146a-5p lead to a significant decrease in dendritic spine density in hippocampal neurons *in vivo* and *in vitro* (Prada et al., 2018).

Conversely, the exosomes derived from M2-MG may present beneficial effects. M2-MG-derived exosomes can attenuate neuronal apoptosis after oxygen-glucose deprivation (OGD) *in vitro* and reduce infarct volume at 3 days of the onset of IS *in vivo* *via* miR-124 and its downstream target ubiquitin-specific protease 14 (Song et al., 2019). BV2 microglial cells polarized by IL-4 can ameliorate ischemic injury by promoting angiogenesis through the secretion of exosomes containing miRNA-26a (Tian et al., 2019). In addition, M2-EVs, enriched in miR-124, can reduce glial scar formation by inhibiting the proliferation of astrocytes and promoting astrocytes’ transition to neural progenitors, and finally improve recovery after stroke (Li Z. et al., 2021).

### Neurons Influence the Function of Microglia

Damaged neurons are not merely passive targets of microglia but rather regulate microglial activity through different signals. CX3CL1/CX3CR1 are the most studied ligands and receptors pairs involved in controlling microglial activation. CX3CL1, which mainly derives from neurons, interacts with CX3CL1R expressed in microglia and directly induced microglial migration, proliferation, and even the ability to eliminate the degenerated neuron debris. CX3CL1 released from injured neurons may signal microglia as a “help me” signal (Suzumura, 2013). Interestingly, dying neurons in the lesioned region release the chemokine CX3CL1, leading to exposure of the “eat-me” signal, which is subsequently recognized by C3aR-expressing microglia, resulting in phagocytosis of living neurons (Surugiu et al., 2019). In contrast to “eat-me” signals, “do not eat me” signals, such as CD47-SIRPα and CD200-CD200R, inhibit microglial phagocytosis (Marinelli et al., 2019; Wang K. et al., 2021).

Damaged neurons also regulate the polarization of microglia. A study (Xing et al., 2014) showed injured neurons could activate microglia into potentially pro-recovery phenotypes *via* lipocalin-2. Lipocalin-2 treatment increase IL-10 release in microglia; meanwhile, conditioned medium from lipocalin-2 treated microglia prevented neuronal death from OGD stress. In line with this, damaged neurons under ischemia stress could release IL-4, which could enhance the expression of the IL-4 receptor on microglia and promote microglia polarization to the M2- phenotype. IL-4 also enhances peroxisome proliferator-activated receptor γ (PPARγ)-dependent phagocytosis of apoptotic neurons (Zhao et al., 2015). *In vitro*, primary neurons in OGD cultures upregulate VEGF release and adding VEGF to microglial cultures partly shifts M2-MG markers (Esposito et al., 2018). In contrast to these studies, Meng and colleagues (Meng et al., 2016) discovered that ischemic neurons could contribute to M1-MG polarization at 24 and 72 h after MCAO by releasing soluble FasL and activating JAK2/STAT3 and NF-κB signaling pathways may be involved.

Neurons critically regulate microglia activity and control inflammation *via* EV-mediated neuron-glia communication. In primary cell culture systems, neurol derived-EVs could improve microglia viability *via* inhibition of apoptosis and reduce the LPS-induced pro-inflammatory response in microglia but increase expression of the anti-inflammatory cytokine, IL-10 (Peng et al., 2021). However, injured neurons show a different effect on microglia activity. Yin et al. (2020) team found scratch-injured neurons PC12-derived exosomes containing miR-21–5p induced M1 microglia polarization. Endangered neurons treated with glutamate could release vesicles containing CCL21 and activate remote microglia *via* the chemokine receptor CXCR3 (de Jong et al., 2005).

### Astrocyte

Astrocytes, as well as microglia, actively control CNS physiology in health and disease (Linnerbauer et al., 2020). Astrocytes undergo a pronounced transformation called “reactive astrogliosis” after brain injury and disease (Liddelow et al., 2017). Reactive astrocytes may be detrimental or beneficial, depending on the heterogeneous types. In one of the first examples of the heterogeneity of reactive astrocytes, A1 and A2 states are identified in LPS injection or ischemic injury *via* MCAO, respectively (Zamanian et al., 2012), which are themselves loosely based on the M1/M2 paradigm. Growing evidence highlights that microglia-astrocyte crosstalk is fundamental to neuronal dysfunctions in CNS disease (Jha et al., 2019; Vainchtein and Molofsky, 2020).

### Microglia Regulate the Activity of Astrocytes

Microglia, which usually react faster than astrocytes to pathological stimuli, induce astrocytes activation and determine the fate of astrocytes, ranging from neuroprotective to neurotoxic (Jha et al., 2019). Either by acute CNS injury or systemic LPS administration, microglial activation can induce A1 astrocytes by secreting IL-1α, TNF-α, and C1q. A1 astrocytes induce neuronal and oligodendrocyte death rather than support neuronal survival and outgrowth (Liddelow and Barres, 2017; Liddelow et al., 2017). SDF1α-CXCR4 signaling has been reported to be involved in the microglia-astrocyte conversation in neuroinflammation. Bezzi et al. (2001) found that autocrine/paracrine TNFα-dependent signaling leads to glutamate release by astrocytes through SDF1α-CXCR4 signaling. Moreover, activated microglia cooperate to enhance the release of TNF-α and further dramatically amplify the TNF-α reaction. Microglia also induce the neuroprotective phenotype of astrocytes and reduce post-stroke inflammation. ZEB1 is a zinc finger-homeodomain transcription factor family member that modulates cell differentiation and specific cellular functions in multiple tissues. After the experimentally induced stroke, ZEB1 is highly expressed in the ischemic cerebral hemisphere, predominantly in microglia. Overexpression of ZEB1 mediated microglial cell responses, followed by inhibition of astrocytic CXCL1 production, resulting in fewer neutrophils entering the brain (Li et al., 2018). In the late phase of IS, astrocytes migrating around BBB will form glial scars to limit the diffusion of inflammatory areas. However, glial scar formation may hinder blood circulation and tissue regeneration in the recovery phases of IS. EVs enriched in miR-124 derived from M2-MG inhibit astrocyte proliferation, thus reducing glial scar formation and promoting recovery after stroke (Li Z. et al., 2021).

### Astrocytes Influence the Behavior of Microglia

Similarly, astrocytes regulate microglial phenotypes and functions through their secreted molecules. Astrocyte-derived chemokines mediate microglial activation. *In vitro* study showed that CCL2, released from TNF-α primed astrocyte, stimulates microglia toward M1 polarization and enhances microglia’s migration ability (He et al., 2016). In addition, the critical role of astrocyte-derived CCL7 in promoting microglia-mediated inflammation was recently demonstrated both *in vivo* and *in vitro* (Xue et al., 2021). In response to stressors, astrocytes can also produce amounts of IL-17A, which contribute to microglia polarization to the M1 phenotype through a signaling pathway with the IL-17A receptor (Das Sarma et al., 2009; Dai et al., 2019; Liu et al., 2019). Notably, astrocytes also participate in a positive feedback loop with microglia that can further exacerbate BBB disruption and neuroinflammation. For instance, pro-inflammatory cytokines such as IL-1β that are released from activated microglia can suppress astrocytic production of sonic hedgehogs and increase secretion of CCL2, CCL20, and CXCL2 (Wang et al., 2014).

Furthermore, astrocytes can attenuate microglial inflammation. For instance, astrocyte derived-exosomes containing miR-873a-5p could significantly inhibit LPS-induced microglial M1-phenotype transformation and the subsequent inflammation through decreased phosphorylation of extracellular regulated protein kinases (ERK) and NF-κB/p65 (Long et al., 2020). Min et al. (2006) treated microglia with the astrocyte culture-conditioned media (ACM) and found ACM suppressed IFN-γ-induced microglial inflammatory responses through the expression of hemeoxygenase-1. Astrocytes in the mixed glial culture stimulated the proliferation of the microglia and M2 polarization, possibly through acquiring the A2 phenotype; both could be converted to M1 microglia and A1 astrocytes under the inflammatory stroke-mimicking environment (Kim and Son, 2021). In addition, astrocytes and neurons combine to provide immunomodulatory cues, repressing primed microglial responses to weak inflammatory stimuli (without affecting maximal responses) and consequently limiting the feedback effects of inflammation on the neurons and astrocytes themselves (Baxter et al., 2021). In addition, IL-10 production by activated microglia stimulates astrocytes to secrete TGF-β, which attenuates microglia activation (Norden et al., 2014), thus forming a negative feedback loop.

### Oligodendrocyte

Oligodendrocytes (OLs), the unique cell type implied in CNS myelination, are sensible to ischemic damage. Swelling of OLs occurred 30 min after arterial occlusion, and a large number of OLs died 3 h after ischemia, which appeared earlier than neuronal death in the ischemic area (Pantoni et al., 1996). Loss of myelin sheaths can impair axon survival, so new oligodendrocyte progenitor cells (OPCs) are required for remyelination. Stroke and experimental ischemia can induce the proliferation and differentiation of OPCs (Zhang et al., 2013). However, most of these OPCs fail to develop into mature OLs, resulting in insufficient remyelination (Goldman and Osorio, 2014; Wang et al., 2020).

Activated microglia regulate the events leading to myelin regeneration (Miron, 2017). The pro-inflammatory factors produced by M1-MG cause OLs’ death. For instance, TNF-α and IFN-γ induce OLs apoptosis and inhibit OPC proliferation and differentiation (Chew et al., 2005; Shi et al., 2015). However, activation of microglia with IL-13 or IL-10 enhances the survival and differentiation effect of OPCs (Miron et al., 2013). Recent studies have shown that TGF-α produced by microglia has a protective effect on OLs against ischemic injury *via* STAT3 signaling (Dai et al., 2020). The work of Xie et al. (2021) revealed that IL-33 treatment reduced the death of OLs and OPCs after MCAO in a microglia/macrophage dependent manner. Combined with their *in vitro* experiment, they concluded IL-33could lead to an anti-inflammatory microglia response, and IL-33-treated microglia could protect OLs and OPCs against stroke (Xie et al., 2021). Moreover, protective microglia-derived EVs enhance the maturation of OPCs, resulting in ameliorated neurological functionality (Lombardi et al., 2019; Raffaele et al., 2021). Minocycline, a member of the tetracycline antibiotic family, is an inhibitor of microglial activation. Inhibiting microglial activation with minocycline attenuated OLs/OPCs damage under hyperoxia and ischemia-hypoxia conditions in neonatal rats (Schmitz et al., 2014). Thus, the effect of microglia on remyelination in stroke is closely related to their functional phenotypes.

### Microvascular Endothelial Cell

Blood-brain barrier (BBB) disruption is a major pathological hallmark in the course of IS (Liu et al., 2018; Shi et al., 2018; Arba et al., 2021). The opening of the BBB progresses from initially reversible (0–24 h) to irreversible (24–72 h) following acute ischemic stroke (Sifat et al., 2017). The primary barrier of BBB is formed by microvascular endothelial cells (EC) with tight junction protein complexes. Upon vascular inflammation, EC can produce lots of factors to trigger microglia prime. For instance, CXCL5 is increased in the cerebrospinal fluid of patients with IS in the first 24 h (Zaremba et al., 2006), and LPS primed EC is the cell source of CXCL5. The pharmacological inhibition of CXCL5 significantly reduces microglial activation. Matrix metalloprotease-3 (MMP-3) is another EC-derived factor that induces microglia activation after spinal cord injury (Lee et al., 2015). Besides, VEGF expressed by EC plays a neuroprotective effect in IS by inhibiting the pro-inflammatory response in microglia *via* downregulated scavenger receptor A (Xu et al., 2017). However, unchallenged endothelial cells showed the opposite performance in microglia activation (Thurgur and Pinteaux, 2019). Resting EC may dampen microglia activation and exert immunosuppressive effects.

Likewise, activated microglia affect BBB integrity targeting on EC. *In vivo* study demonstrated that perivascular microglia may directly supply tight junction protein (TJP) claudin-5 to the endothelium and contact EC to maintain BBB integrity. While sustained inflammation triggers activated microglia to impair BBB function by engulfing astrocytic end-feet (Haruwaka et al., 2019). Cytokines and chemokines derived from activated microglia are the primary mediators involved in inflammation and BBB injury. In a co-culture system of microglia and microvascular endothelial cells, studies show LPS-stimulated microglia (LPS-MG) induced BBB collapse *via* TNF-α, IL-1β, IL-1α, and MIP-1α (Nishioku et al., 2010; Shigemoto-Mogami et al., 2018). It is noteworthy LPS-MG triggered astrocytes could produce a higher concentration of these cytokine/chemokines (Shigemoto-Mogami et al., 2018). Besides, TNF-α secreted by microglia may also induce EC necroptosis, which contributes to BBB breakdown in IS (Chen et al., 2019). Likewise, IL-1β expressed by microglia could induce downregulation of TJP ZO-1/occluding and increase BBB permeability *in vitro* (Kangwantas et al., 2016). In addition, immune cells are trafficked into brain parenchyma by chemokines (i.e., CCL2, CXCL10) secreted by M1-MG, which generate reactive oxygen species and thus exacerbate BBB damage (Thurgur and Pinteaux, 2019; Ronaldson and Davis, 2020). Recently, Xie et al. (2020) reported that the exosomes shuttled miR-424-5p from OGD activated microglia significantly cause cell injury and permeability of EC, and the inhibition of miR-424-5p markedly reduced neurological dysfunctions and endothelial cell injury induced by MCAO.

## The Communication Between Microglia and CNS-Infiltrating Cells

**Figure 2:**
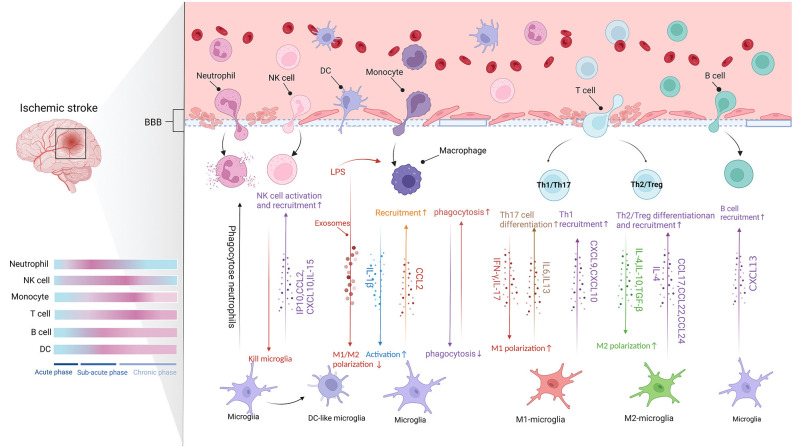
The communication between microglia and CNS-infiltrating cells (Figure created with BioRender.com). Following BBB destruction, peripheral immune cells infiltrate into brain parenchyma and work with microglia to damage or protect the brain tissue. The migration of leukocytes to ischemic lesions presents a time scale and they act in different stages of stroke (Blue represents baseline conditions, whereas pink indicates peak modifications of the immune cells. Acute phase: up to 8 h after stroke. Subacute phase: 8–48 h after stroke. Chronic phase: over 48 h after stroke). Microglia release chemokines to promote the infiltration of peripheral immune cells. Moreover, different signals crosstalk between different subtypes of peripheral immune cells and different microglia phenotypes affect pro- or anti-inflammatory function.

Numerous peripheral immune cells sequentially infiltrate into the ischemic hemisphere after acute ischemic stroke (Qiu et al., 2021). Microglia can regulate the recruitment, extravasation, and function of peripheral immune cells. For instance, microglia release a variety of chemokines to stimulate neutrophils, monocytes, and lymphocytes in the injured area after ischemic injury. Microglia also regulate the pro-inflammatory or anti-inflammatory state of infiltrating peripheral immune cells after IS. Conversely, invasive peripheral immune cells also affect the function of microglia in IS (Qiu et al., 2021). The timescale and the communication between microglia and the infiltrating cells in the brain are shown in Figure 2.

### Neutrophil

Neutrophils are the first peripheral immune cells attracted to the brain, where they are detected in the microvascular within the 1st h and peak at 1 to 3 days after experimental stroke (Gelderblom et al., 2009; Gronberg et al., 2013). The infiltration of neutrophils in the ischemic hemisphere increased significantly after 3 h, reaching a peak after 24 h, followed by a steady dissipation over 7 days (Gelderblom et al., 2009). Neutrophils can exert detrimental effects already from the vessel wall, inducing a no-reflow phenomenon, damaging BBB, and promoting clot formation (Planas, 2018). Also, studies found neutrophils in the ischemic brain parenchyma using models of MCAO. The invading neutrophils can accelerate neuronal damage in ischemic conditions and enlarge infarct lesions (Neumann et al., 2008; Perez-de-Puig et al., 2015; Otxoa-de-Amezaga et al., 2019). Both neutrophils and microglia can respond rapidly after cerebral ischemia. Evidence demonstrated that microglia could counteract the neurotoxicity of neutrophils by phagocytosis of apoptotic and viable, motile neutrophils in cell culture and within brain slices (Neumann et al., 2008). More neutrophils are gathered in the ischemic core where local microglia cell loss and dystrophy. In contrast, fewer neutrophils at the periphery of infarction are associated with more reactive microglia at 1 day to 4 days after MCAO (Otxoa-de-Amezaga et al., 2019).

Interestingly, neutrophils may protect neuron damage against ischemic injury *via* the anti-inflammatory phenotype N2 expressing Ym1 and CD206 (Hou et al., 2019). Neutrophils can reprogram to N2 phenotype in brain inflammation which is modulated by PPAR-γ activation or by TLR activation. The N2 polarization of neutrophils promoted the engulfment of neutrophils by microglia/macrophages, which led to reductions in brain edema and infarct volume (Cuartero et al., 2013; Garcia-Culebras et al., 2019). Thus, neutrophils may also have a dual function in IS by amplifying inflammatory response or the resolution of inflammation.

### NK Cell

NK cells are subsets of cytotoxic lymphocytes that respond quickly after activation and kill cells by releasing cytokines and chemokines without pre-stimulation from other cells (Abel et al., 2018). After BBB destruction, NK cells infiltrate into brain parenchyma like other peripheral immune cells, exhibiting dynamic and spatiotemporal characteristics. Zhang et al. (2014) observed NK cells in ischemic patients’ brain tissues in 2 to 5 days and found the number of NK cells in the ischemic hemispheres was higher than those in the non-ischemic hemispheres. In addition, the infiltration of NK cells into the ischemic infarct region reached its highest levels 12 h after ischemia (Zhang et al., 2014). Moreover, kinetic experiments showed that NK cells accumulate in the brain as early as 3 h after tMCAO and peak on day 3 (Gan et al., 2014).

Chemotaxis and infiltrates of NK cells into the brain may be associated with microglia. During the IS, microglia are activated and release a large amount of IP-10. IP-10 belongs to the chemokine-like factor superfamily, which has chemotactic activity against natural killer cells. NK cells were recruited to the ischemic region through the IP-10/CXCR3 and CX3CL1/CX3CR1 axis (Zhang et al., 2014). Additionally, NK cells are also recruited to the CNS by chemokines such as CCL2 and CXCL10 produced by microglia (Earls and Lee, 2020).

NK cells control CNS inflammation by killing microglia. A study showed that NK cells only kill resting microglia *via* NKG2D- and NKp46-mediated recognition (Lünemann et al., 2008). In addition, NK cells depletion induces a protective microglia phenotype in the context of glioma and Alzheimer’s disease (Garofalo et al., 2020; Zhang Y. et al., 2020). On the contrary, microglia also affect the function of NK cells. IL-15 derived from microglia promote the levels and activation of NK cells, thereby enhancing the destruction of BBB in IS (Lee et al., 2018).

### Monocyte/Macrophage

Monocytes are the precursors of macrophages. The ischemic environment promotes the differentiation of monocytes into macrophages. The immature monocytes infiltrate into ischemic tissue and gradually differentiate into monocytes-derived macrophages (MDMs) after IS (Murray and Wynn, 2011). Peripheral monocytes infiltrate the lesion within 24 h after IS, but only a small number of monocytes infiltrated into the lesions within 2 days after cerebral ischemia (Wattananit et al., 2016). Next, the number of monocytes entering lesions increased significantly and peaked at 3–7 days after ischemia, then gradually decreased. On the 28th day after cerebral infarction, MDMs were still found in the ischemic core (Garcia-Bonilla et al., 2016). After tMCAO, monocytes infiltration was found in the peri-infarction and the core of the infarction. However, after pMCAO, monocytes’ initial infiltration occurs primarily in the infarct core (Zarruk et al., 2018; Han et al., 2020).

MDMs are most abundantly recruited to the injured brain during the subacute and chronic phases of IS. In the chronic phase of ischemic stroke, recovery mechanisms such as neuronal remodeling are activated in the brain (ElAli and LeBlanc, 2016). The above conclusions suggest that MDMs may play a neuroprotective role. The findings of Wattananit et al. (2016) revealed that infiltrating MDMs contribute to the long-term functional recovery of IS, and this protective function could be attributed to their anti-inflammatory activity. There is evidence that MDMs are more likely than microglia to polarize to the M2 phenotype and exert their anti-inflammatory function (Kronenberg et al., 2018), and have a more phagocytic function than microglia (Ritzel et al., 2015).

Infiltrated MDMs affected the behavior of resident microglia. After a photothrombotic stroke (PT), monocyte infiltration decreased in *Cxcr4* cKO mice, associated with reduced microglial proliferation, suggesting that MDMs might promote repopulation of the infarct by microglia. In support of this, RNA-seq indicated that microglia-activating mediators such as IL-1β are mainly provided by MDMs after PT. Moreover, Gene Ontology (GO) term enrichment analysis for microglia differentially expressed genes (DEGs) suggested that the genes associated with microglial activation were overexpressed, while the genes involved in homeostatic cellular metabolism were downregulated. In addition, macrophage depletion reduced the microglia activation in both the ipsilateral cortex and striatum, thereby facilitating neurological recovery after MCAO (Ma et al., 2016). Moreover, exosomes produced by LPS-stimulated macrophages displayed the anti-inflammatory and neuroprotective effects regulating the transformation of microglia from M1 to M2 (Zheng et al., 2019). Perego et al. (2016) reported that the depletion of MDMs increases microglia’s M1/M2 polarization ratio and exacerbates ischemic stroke injury. Additionally, the inflammatory cascades in microglia are suppressed in the presence of macrophages. Pro-inflammatory cytokines, such as IL-1β, TNF-α, and IL-6, were significantly suppressed in the presence of macrophages (Greenhalgh et al., 2018). The phagocytic functions of microglia and MDMs can interact with each other. Furthermore, using co-cultures of microglia and macrophages, the data showed that the presence of macrophages suppressed myelin uptake by microglia. In contrast, the presence of microglia enhanced myelin uptake by blood-derived monocytes. And the result indicated that there was direct communication between the two cell types, which affected phagocytosis differently (Greenhalgh et al., 2018).

### T Cell

As mentioned above, microglia rapidly activate after IS onset and promote the invasion of peripheral immune cells into the brain by cytokines and chemokines (Xu et al., 2020). In animal models, T cells, the prominent effector lymphocytes in the peripheral immune system, appear in brain parenchyma as early as the first 24 h after MCAO and peak at 3 to 5 days or 7 days after tMCAO or pMCAO respective (Gelderblom et al., 2009; Gronberg et al., 2013). T-cell activity persists for at least 1 month following the experiment stroke (Xie et al., 2019), and in patients with IS, the increase in T-cell numbers lasts up to 3 months (Heindl et al., 2021).

T cells play different roles in the ischemic brain related to functional subtypes. T cells divide into αβ subset and γδ subset. αβ subset further divides into three subtypes, including CD8^+^ cytotoxic *T* lymphocytes (CTL), CD4^+^ T helper cells (Th), and regulatory T cells (Tregs; Hedrick, 2002). CTL may be recruited as early as 3 h after stroke onset (Chu et al., 2014) and then exacerbate brain damage by killing target cells directly or indirectly (Mracsko et al., 2014). γδT cells are found in infarct boundary zones, and their counts increase from day 1 to day 6 after IS and peak on day 3 (Shichita et al., 2009). γδT cells, but not Th17 cells, are the major source of IL-17 in the acute phase of IS, contributing to mononuclear cells infiltration (Shichita et al., 2009). In addition, a small number of double-negative T cells (CD3^+^CD4^−^CD8^−^T) were also found within the ischemic penumbra. The infiltrating double-negative T cells could promote M1-MG transition through FasL/PTPN2/TNF-α signaling pathway and thus aggravate ischemic brain injury (Meng et al., 2019). Th cells, functionally, are classified as Th1, Th2, and Th17 cells based on their cytokine secretion profiles (Stockinger et al., 2006). The complex role of Th cells themselves and their dialogue with microglia in the context of stroke will be clarified in detail below.

### Th1/Th17

The initial infiltration of Th1 cells occurs in the meninges within 24 h after IS (Gronberg et al., 2013) and follows a massive appearance in the peri-infarct and infarct area at 7 days (Ortolano et al., 2010). The cells do not kill cells directly but help activate other immune cells, including CTL (Theodorou et al., 2008). Th1 cells together with M1-MG produce high levels of pro-inflammatory cytokines, iNOS, and neurotoxic substances that induce an inflammatory response and accelerate brain injury after IS (Korhonen et al., 2015). Studies have shown that Th1 cells can produce soluble cytokines, such as IFN-γ, which transform the microglia to M1-phenotype and thereby increase secondary ischemic damage (Chabot et al., 2001). M1-MG also expresses chemokines such as CXCL9 and CXCL10, which recruit Th1 cells for inflammatory response (Mantovani et al., 2004). Like Th1 cells, Th17 cells interact with M1-MG to play a pro-inflammatory role (Klebe et al., 2015). Th17 cells may promote microglia polarization to the M1-phenotype by selective IL17, thereby amplifying inflammatory effects (Denning et al., 2007). While, M1-MG induces Th17 cell differentiation through secretion of IL-6 and IL-23, and thus promotes the immune response together (Wang et al., 2015).

Thus, Th1/Th17 cells can promote neuroinflammation by secreting pro-inflammatory cytokines and can also enhance M1-MG-mediated neurotoxic responses.

### Th2

Th2 cells appear near the leptomeninges at 24 h after IS (Gronberg et al., 2013). Torres et al. (2004) suggested that expression of Th2-associated cytokines occurs only upon stimulation of cytokines IL-12 or IL-4 and endogenous produced IFN-γ and IL-4. Studies show that M2 macrophages-secreted cytokines stimulate Th2 cells, which produce high levels of IL-4 and IL-10, and further facilitate M2-MG polarization (Korhonen et al., 2015; Xiong et al., 2015; Zhao et al., 2017). Moreover, it has been shown that both M2-MG and Th2 cells reduced the infarct area and promoted the neuronal regeneration process (Korhonen et al., 2015). Meanwhile, M2-MG drives Th2 cell recruitment and polarization by secreting cytokines and chemokines, such as IL-4, CCL17, CCL22, and CCL24, thus amplifying the type 2 response and having a protective effect against post-stroke inflammation (Biswas and Mantovani, 2010).

### Treg

Regulatory T cells (Tregs), a subset of T cells characterized by the transcription factor Foxp3, account for 5%–10% of the peripheral CD4^+^ T cell population (Chen et al., 2003). Several studies observed that Tregs in and around the infarct area increase from the first week after IS and remain at high levels for 2 months (Gelderblom et al., 2009; Stubbe et al., 2013; Ito et al., 2019). Contrary, Kleinschnitz et al. (2013) found a marked increase of Foxp3^+^ Tregs in the brain within 24 h after tMCAO, but predominantly in the cerebral vasculature. Tregs play a double-edged role in IS, but primarily with a neuroprotective effect (Wang H. et al., 2021). Brain Tregs can modulate the microglia/macrophages polarization toward M2-type in cerebral ischemia by secreting IL-10 and TGF-β, therefore protecting against secondary brain damage (Liesz et al., 2009; Liu et al., 2011). In addition, Tregs promote microglia-mediated tissue repair through secreting osteopontin (Shi L. et al., 2021). In turn, M2-MG can also promote the differentiation of Tregs to alleviate neuroinflammation (Brea et al., 2014; Shu et al., 2019).

In conclusion, these findings show that Th2/ Treg cells exert a neuroprotective role related to reducing the inflammatory response and promoting the M2-MG response.

### B Cell

Minimal amounts of B cells can normally be detected in the brain but are transported in large quantities to CNS during injury or disease (Anthony et al., 2003; Funaro et al., 2016). Li S. et al. (2021) showed a significant increase in the proportion of B cells among lymphocytes in patients with acute ischemic stroke that persisted for up to 3 months. In addition, the percentage of B cells within lymphocytes at 1 month inversely correlated with the admission National Institute of Health Stroke Scale (NIHSS) score (Li S. et al., 2021). On the contrary, regulatory B cells (Bregs) that secret IL-10 are thought to be protective cells after stroke (Berchtold et al., 2020). The percentage of Bregs within lymphocytes dramatically increased in stroke patients (Li S. et al., 2021). Infiltration data from animal models showed that B cells increase by three- to four-fold over sham levels at 3 and 24 h after pMCAO. However, B cells increased two-fold at 24 h after tMCAO (Chu et al., 2014). The infiltration of B cells into the ischemic brain takes several weeks. B cells appear in the lesion 7 weeks following IS in C57BL/6 mice (Doyle and Buckwalter, 2017), and only a small part of Bregs accumulate within 1–2 days after stroke (Prinz and Priller, 2017).

The delayed infiltration implied that B cells might play an important role during the chronic phase of stroke. Activated B cells from both humans and mice have been shown to produce neurotrophic growth factors, such as BDNF, to promote the survival and differentiation of neuronal populations during CNS injury (Kerschensteiner et al., 1999; Edling et al., 2004; Fauchais et al., 2008). Moreover, the addition of BDNF stimulates microglia proliferation (Harley et al., 2021). However, as there are various cellular sources of BDNF in the brain, the significance of its expression by microglia *in vivo* remained obscure.

B cells may affect the function of microglia. The lack of B cells in μMT^−/−^ mice further permitted significant increases in the absolute number of microglia in the ipsilateral hemisphere of MCAO mice (Ren et al., 2011). Several studies have observed B cells activated by CNS-specific antigens in the spleen 4 days after stroke. Meanwhile, splenic B cells showed a broad range of microtubule-associated protein 2 (MAP2) peptide responses (Ortega et al., 2015). Given that MAP2 is associated with infarction volume reduction and functional recovery (Hsu and Jones, 2006; Zhang et al., 2012), B cells may play a role in neuroprotection and post-stroke repair. In addition, some researches showed that microglia could differentiate into MAP2-positive cells, and these cells possess properties of functional neurons (Matsuda et al., 2008). Microglia can also affect B cells. Research on other inflammatory conditions of the CNS shows that microglia could potentially contribute to B cell infiltration through CXCL13 production (Berchtold et al., 2020), and CXCL13 is a specific chemokine for B cell homing.

### Dendritic Cell

Dendritic cells (DCs) are critical components of the innate immune and adaptive immune response. DCs detect and accumulate foreign antigens and present antigens to naive T cells (Zhang H. et al., 2020). No increase in DCs was observed at the early stages of tMCAO (6 h) and pMCAO (3 h). At 24 h after MCAO, DCs accumulate in ischemic hemispheres and form clusters in the region surrounding cerebral infarction (Chu et al., 2014). In addition, DCs located primarily in the marginal region of the infarction where T lymphocytes congregate and contain high levels of major histocompatibility class II (MHC II) and the co-stimulatory molecule CD80. The location of DCs may be related to their role in presenting antigens for T cells. At 72 h after MCAO reperfusion, the DC appears in the core and boundary of the infarction, although the cells in the core take an ovate form compared to the branch cells at the infarct boundary (Felger et al., 2010).

Studies have shown that microglia can be converted to DC-like phenotypes that express the DC surface marker CD11c after cerebral ischemia/reperfusion (Butovsky et al., 2006). Studies have also shown that microglia can be converted to DC-like phenotypes *in vitro* (Rodriguez-Gomez et al., 2020). IFN-γ/c-myc/ERK signaling pathway regulated the transformation of microglia into DC-like cells (Zhang H. et al., 2020). In addition, Santambrogio et al. found that the microglia might be uncommitted myeloid progenitors of immature DCs (Santambrogio et al., 2001). Moreover, microglial SIRPα inhibited the induction of CD11c^+^ microglia, which may accelerate the repair of damaged white matter (Sato-Hashimoto et al., 2019). Of note, other researchers hold different views. Dando et al. (2016) challenged the notion that CD11c-eYFP^+^ cells are DCs or their immature precursors. They thought these DC-like phenotypes were most likely subpopulations of microglia. Based on the current findings, researchers still need to pay attention to the relationship between peripheral DCs and microglia.

## Conclusion

In recent years, there has been a fast-growing interest in microglia, as they are increasingly implicated in various CNS disorders and diseases. Microglia exhibit diversity and molecular heterogeneity in the context of different stimuli. In addition to the most simplistic M1/M2 classification, additional microglia populations have been reported, such as disease-associated microglia (DAM), lipid-droplet-accumulating microglia (LDAM), proliferative zone associated microglia (PAM), and glioma-associated microglia (GAM) states (Wright-Jin and Gutmann, 2019; Marschallinger et al., 2020), and these findings are attributed to multi-omics technologies and single-cell RNA sequencing. However, whether these differences in molecular heterogeneity affect the role of microglia in the development of IS needs to be further investigated.

Microglia, the immune sentinels of the CNS, are the first to be activated to phagocytose damaged neurons or cellular debris in response to ischemic injury. Besides, activated microglia crosstalk to other resident cells in the brain, including neurons, astrocytes, and microvascular endothelial cells, either directly or indirectly. These cells interact with each other and even form feedback loops to participate in subsequent pathological events of stroke. Many cellular signals are involved in microglial cell conversations, and it is worth mentioning that the role of extracellular vesicles in their communication has received increasing attention. The cascade of post-stroke inflammatory signals leads to a disruption of the BBB, followed by a massive infiltration of peripheral immune cells participating in the secondary progression of ischemic brain injury. Microglia monitor these infiltrating immune cells on the one hand, and on the other hand, the activity and function of microglia are reshaped by these cells.

In conclusion, intercellular communication centered on microglia plays a neuroprotective or neurotoxic role in the process of IS. This dual role is related to the functional subtypes of the cells and the stage of the stroke course. Since microglia may be the integrators of many cellular signals, treatment strategies that interrupt microglia function could evolve into future adjuvant stroke therapies. However, the translation of global microglia inhibition strategies (i.e., minocycline, PLX3397) from animal models to humans is yet to yield a positive result to date (Wright-Jin and Gutmann, 2019). Therefore, these future approaches may involve targeting specific microglia subpopulations or functions or indirectly regulating microglia function through other cells (Herisson et al., 2018). Based on the results of clinical translational trials, the study of multi-target neuroprotective agents should be a hot spot for future research in stroke therapy (Paul and Candelario-Jalil, 2021).

## Author Contributions

Conceptualization, writing—review and editing: YZ and SX. Writing—original draft preparation: YZ, LL, RF, XS, JL, and YJ. Supervision: SX. All authors contributed to the article and approved the submitted version.

## Funding

This work was supported by the National Natural Science Foundation of China (No. 81774059), the Tianjin Natural Science Foundation (No. 19JCZDJC37100), and the School of Integrative Medicine Foundation in Tianjin University of Traditional Chinese Medicine (No. ZXYKYQDLX202001).

## Conflict of Interest

The authors declare that the research was conducted in the absence of any commercial or financial relationships that could be construed as a potential conflict of interest.

## Publisher’s Note

All claims expressed in this article are solely those of the authors and do not necessarily represent those of their affiliated organizations, or those of the publisher, the editors and the reviewers. Any product that may be evaluated in this article, or claim that may be made by its manufacturer, is not guaranteed or endorsed by the publisher.
